# Printing of Crumpled CVD Graphene via Blister-Based Laser-Induced Forward Transfer

**DOI:** 10.3390/nano10061103

**Published:** 2020-06-02

**Authors:** Maxim S. Komlenok, Pavel A. Pivovarov, Margarita A. Dezhkina, Maxim G. Rybin, Sergey S. Savin, Elena D. Obraztsova, Vitaly I. Konov

**Affiliations:** 1 Prokhorov General Physics Institute of the Russian Academy of Sciences, Vavilova Street 38, 119991 Moscow, Russia; p_pivovarov@hotmail.com (P.A.P.); m.a.dezhkina@gmail.com (M.A.D.); rybmaxim@gmail.com (M.G.R.); elobr@kapella.gpi.ru (E.D.O.); vik@nsc.gpi.ru (V.I.K.); 2 MIREA—Russian Technological University, Vernadsky Avenue 78, 119454 Moscow, Russia; savin@mirea.ru

**Keywords:** laser-induced forward transfer, graphene, laser processing, laser ablation, Raman spectroscopy

## Abstract

The patterning and transfer of a two-dimensional graphene film without damaging its original structure is an urgent and difficult task. For this purpose, we propose the use of the blister-based laser-induced forward transfer (BB-LIFT), which has proven itself in the transfer of such delicate materials. The ease of implementation of laser techniques reduces the number of intermediate manipulations with a graphene film, increasing its safety. The work demonstrates the promise of BB-LIFT of single-layer graphene from a metal surface to a SiO_2_/Si substrate. The effect of the parameters of this method on the structure of transferred graphene islands is investigated. The relevance of reducing the distance between irradiating and receiving substrates for the transfer of free-lying graphene is demonstrated. The reasons for the damage to the integrity of the carbon film observed in the experiments are discussed. The preservation of the original crystal structure of transferred graphene is confirmed by Raman spectroscopy.

## 1. Introduction

The use of graphene in optics and electronics requires transferring the monolayer carbon material to an arbitrary, usually dielectric, substrate and forming the necessary pattern from it; for example, the channel of the transistor [1,2]. The standard procedure for the fabrication of a graphene channel for a transistor is a multi-stage electron-beam lithography process [3,4,5]. Each of these stages consists of several steps of covering graphene by the polymer and its further removal. Such multistage processes lead to a significant decrease in the quality of the initially synthesized graphene on copper foil. Laser-induced forward transfer (LIFT) is a more promising method for this purpose due to the wide range of variable parameters (wavelength, pulse duration, energy, and focusing spot) of modern laser systems and their stability, which allow the transfer of materials in a liquid or solid state of aggregation [6,7,8,9,10,11,12,13]. During the LIFT procedure, the laser pulse heats the absorbing layer covering the transparent donor substrate. Local heating leads to the partial evaporation of the absorbing layer and an increase in pressure, which results in the ejection of material from the site of the donor substrate facing the receiving sample–acceptor. Different variations of this technique may be applied depending on the task and the transferred material. Moreover, this method has already been used for graphene transferring [14]. In this case, the triazene film was used as the material absorbing laser radiation, and the graphene film was embedded in a polymethylmethacrylate (PMMA) matrix, which was chemically removed with the remains of the absorbing polymer after the transfer. Here, we propose another modification of this technique with the use of metal film instead of the triazene for the absorption of laser radiation. Such a metal layer protects the transferred material both from overheating and from the photo action of laser radiation and creates the conditions for its transfer. Short-pulse laser heating causes the surface ablation of a thin metal layer on the boundary with a transparent substrate, resulting in a pressure increase and blister formation, the so-called blister-based LIFT (BB-LIFT) [13,15,16,17], which leads to the release of the transferred material in the direction of the acceptor surface. This configuration allows the transfer of free-lying graphene and avoids the use of a PMMA matrix. The need for the subsequent removal of the latter greatly complicates the procedure for the transfer of graphene and leads to additional damage to the carbon film. Recently, we used this approach for the transfer of pure single-walled carbon nanotubes (SWCNTs) [18] and graphene film [19]. However, the generated impulse by the blistering of the 420 nm-thick metal (Al) film used in the previous experiments was not enough to push the light graphene film in comparison with a thick layer of SWCNTs. As a result, the graphene film was incompletely removed from the donor surface and was transferred in the form of individual crumpled flakes of micron and submicron size. These transfer features are associated with a low value of the ablation threshold of the metal layer at a certain metal thickness. The working range of the laser fluence should provide enough impulse for the ejection of the transferred material on the one hand and should, on the other hand, not cause a rupture of the metal film and an ablative metal spatter towards the acceptor substrate. For this reason, the thickness of the aluminum layer should be increased.

Here, we report on the results of the BB-LIFT of the fragments of pure graphene film. The effect of the thickness of the absorbing metal layer and the gap between the donor and acceptor substrates on the quality of the transfer is analyzed. Possible changes in the internal structure and morphology are controlled by Raman spectroscopy and scanning electron microscopy (SEM), respectively.

## 2. Materials and Methods

Experimental samples, the so-called donors, were prepared in two stages. First, to absorb the energy of the pulsed laser radiation, an aluminum film was deposited on the surface of a polished quartz plate. The metal film deposition was performed in a vacuum chamber at a pressure of 10^−5^ mbar by the heating aluminum foil in a molybdenum boat to 900 °C with a current passing through it. Then, a polycrystalline graphene film grown on copper foil by chemical vapor deposition (CVD) was transferred to the surface of the aluminum film by a standard wet method using PMMA as a supporting polymer, which was chemically removed after the transfer [20,21,22]. Thus, a donor sample was obtained, consisting of a transparent quartz substrate coated with a radiation-absorbing metal layer with a free-lying, mainly single-layer, graphene film on its surface.

**Figure 1:**
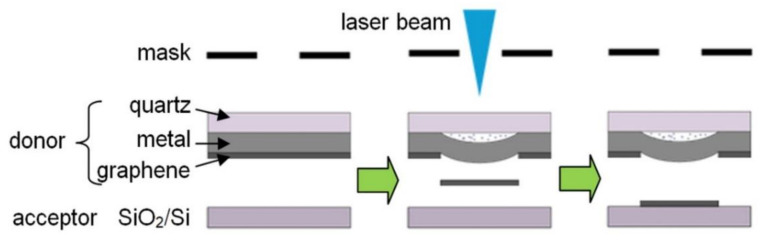
Sketch of the BB-LIFT technique applied to transfer the graphene film.

The BB-LIFT procedure applied to the donor sample containing a graphene film is illustrated in Figure 1. The radiation of the KrF excimer laser CL-7100 (Optosystems Ltd., Troitsk, Moscow, Russia, wavelength 248 nm, pulse duration 20 ns) was directed vertically downward and focused on the surface of the aluminum absorber layer through a square mask (1200 × 1200 mm^2^) to cut out the central homogeneous part of the laser beam. In such a projection scheme, the image of the mask was reduced 20 times into a 60 × 60 μm^2^ spot. In our experiments, the laser fluence in the irradiated spot was varied discretely from 1.2 to 3 J/cm^2^.

Silicon substrates with a 90 nm-thick silicon dioxide (SiO_2_) surface layer were chosen as receiving substrates, the so-called acceptor, which made it possible to control the transfer of the graphene film on the substrate surface by optical methods [23]. The acceptor substrate was placed at a distance of 50 or 0 μm from the donor in two sets of experiments. In the case of zero distance, the acceptor was placed in contact with the donor, but due to the imperfect flatness of the two samples a small gap appeared between them.

Single-layer polycrystalline graphene films of high quality were selected for the experiments. The quality of the films before and after laser irradiation, both on the initial and receiving substrates, was controlled using Raman spectroscopy (Horiba, Paris, France, LabRAM HR Evolution spectrometer with λ = 532 nm) and SEM (TESCAN Mira 3, Brno, Czech Republic). The surface morphology was also analyzed using an Axiotech 25HD optical microscope (Carl Zeiss, Jena GmbH, Germany).

## 3. Results and Discussion

**Figure 2:**
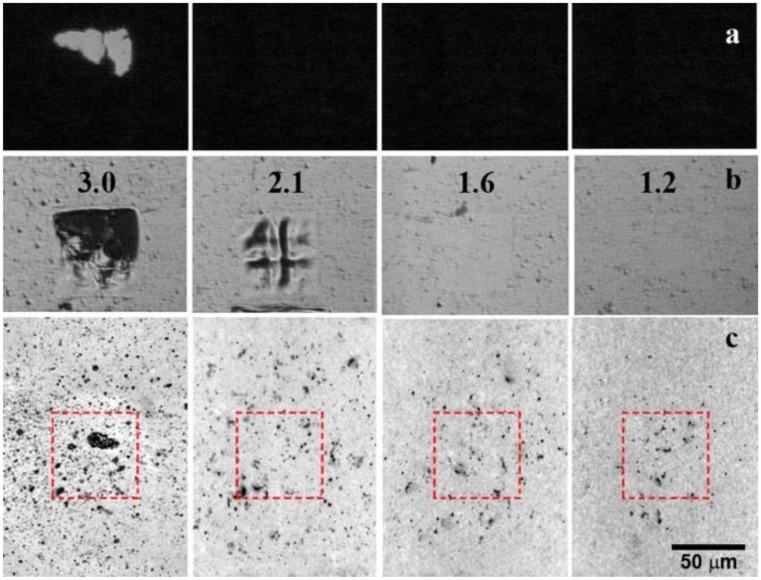
Optical images of the donor substrate covered by the graphene film in the (**a**) transmission and (**b**) reflection modes, and the (**c**) acceptor substrate after the BB-LIFT procedure with a 50 μm gap between substrates. Numbers correspond to the laser fluence in J/cm^2^ used for the transfer.

In this work, we increase the thickness of absorbing aluminum film from 420 to 1900 nm, which leads to an increase in its ablation threshold from 0.8 ± 0.1 to 2.8 ± 0.2 J/cm^2^. In this case, the graphene film starts to transfer after the irradiation with a laser fluence exceeding the value of 1.2 ± 0.1 J/cm^2^. Therefore, the working range of the laser fluence changes from 0.2 ÷ 0.8 to 1.2 ÷ 2.8 J/cm^2^. The distance between the donor and acceptor substrates remains the same as in previous experiments [19] and amounts to 50 μm. The result of BB-LIFT of single-layer graphene film is shown in Figure 2. Optical images of the donor substrate in the transmission and reflection modes after the irradiation with different laser fluences are presented in Figure 2a,b, respectively. Optical images of the acceptor substrate surface are shown in Figure 2c in a mirror image for an easy comparison of the resulting print on the acceptor with the donor. A noticeable amount of the transferred material is observed on the acceptor surface (dark islands in Figure 2c) at all laser fluencies. It is seen that after the laser exposure and transfer, the polycrystalline graphene film breaks into separate micron-sized flakes. With an increasing fluence, the transfer area spreads over the surface of the acceptor substrate beyond the irradiation zone indicated by the dashed red line. This result correlates with the dependence of the scattering angle on the laser fluence observed after the break-up of the diamond layer into micrometer-scale clusters during its BB-LIFT process [13]. When the laser fluence reaches the value of 3 J/cm^2^, a noticeable change in the amount of transferred material onto the acceptor surface is observed, which is associated with the beginning of the ablative metal transfer. It is indicated by the metal rupture that can be observed in Figure 2a, as well as the presence of relatively large metal areas and drops on the receiving substrate (Figure 2c), which clearly differ from the islands of the transferred carbon film.

**Figure 3:**
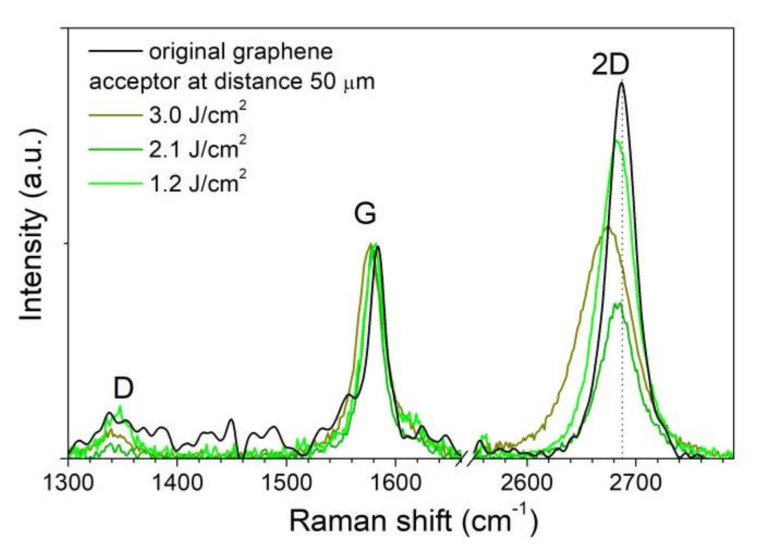
Raman spectra of the graphene films, recorded from the initial film on the donor substrate and areas on the acceptor substrate placed at a distance of 50 μm from the donor.

The Raman spectroscopy analysis of islands of the transferred material shows that graphene is transferred by separate micron-sized flakes to the acceptor substrate with the preservation of the initial crystal structure (sp^2^ hybridization) of single-layer graphene if the laser fluence exceeds the value of 1.2 ± 0.1 J/cm^2^. Figure 3 shows the Raman spectrum of the initial graphene film (black line) on the aluminum surface of the donor substrate and the spectra of material on the acceptor surface transferred with three values of the laser fluence (green lines in Figure 3). The signal-to-noise ratio is higher for the original film because the spectrum was recorded from the graphene on the aluminum film on the donor surface in contrast to the spectra recorded from the silicon acceptor substrate. Distinctive graphene peaks at a Raman shift of 1580 cm^−1^ (peak G) and 2670 cm^−1^ (peak 2D) are detected for the original film and transferred flakes. The width of the 2D peak is about 32 cm^−1^ in almost all regions, which indicates the transfer of single-layer graphene [24]. In the spectra of the transferred graphene flakes, a noticeable shift of the 2D peak towards a lower wavenumber is observed, which indicates an increase in stress in the transferred material [25]. The increase in the laser fluence leads to a stronger shift of the 2D peak and, therefore, to an increase in stress in graphene flakes on the acceptor substrate. The observed decrease in the intensity of the 2D peak relative to the G peak and its broadening with an increase in the laser fluence can indicate the partial oxidation of the carbon film [26]. The appearance of the D peak at 1345 cm^−1^ corresponds to the presence of defects in the carbon material. To analyze the defect formation in the transferred graphene material, we calculate the Id/Ig ratio of the intensities of the D and G peaks in the initial film and transferred graphene fragments. After the transfer, the Id/Ig ratio increases from 0.07 to a maximum of 0.18, which corresponds to a change in the distance between defects from 38 to 24 nm [27].

**Figure 4:**
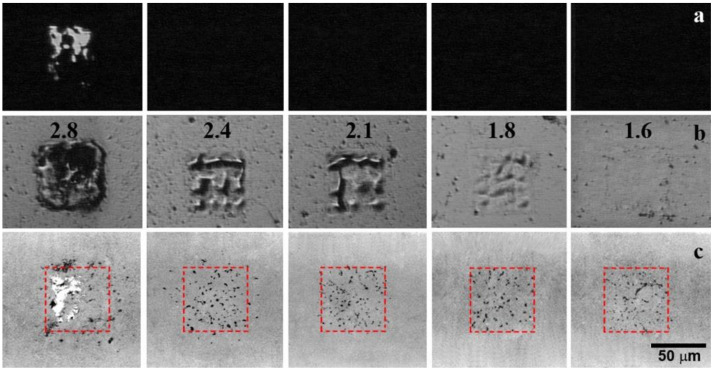
Optical images of the donor substrate covered by the graphene film in the (**a**) transmission and (**b**) reflection modes, and the (**c**) acceptor substrate after the BB-LIFT procedure with no gap between substrates. Numbers correspond to the laser fluence in J/cm^2^ used for the transfer.

The Raman spectroscopy analysis of the donor surface in the irradiated areas after the transfer shows a complete absence of graphene in them at a laser fluence higher than 1.6 ± 0.1 J/cm^2^, which was not previously observed in experiments with a thin metal film at a fluence value reaching the threshold of metal ablation [19]. Thus, an increase in the thickness of the metal layer allowed us to increase the efficiency of removal of transferred graphene from the donor to acceptor surfaces without signs of the metal transfer. However, the nature of the transfer remains the same. Multiple break-ups of the original film, the transfer of micrometer-scale islands of the graphene film, and its crumpling after the transfer is observed. A possible cause of the film crumpling is the free flight of graphene from the donor to the silicon substrate. It is known that a two-dimensional film in a free state tends to reduce the surface energy, which leads to the appearance of bends and the transition to a 3D structure. Therefore, the air gap of 50 μm between the donor and acceptor substrates was removed; and the acceptor was placed in contact with the donor. Besides, by reducing the distance to the receiving substrate, the lateral spread of the transferred material should be reduced. Figure 4 shows optical images of the surfaces of the donor and acceptor after a laser transfer performed with no gap between them. Despite the obvious similarity of the optical images of the donor surfaces in Figure 2a,b and Figure 4a,b after the laser irradiation, the absence of an air gap between the substrates leads to a noticeable change in the morphology of the obtained patterns on the silicon acceptor substrate. The patterns, shown in Figure 4c, demonstrate the absence of a noticeable lateral spread of the transferred material at all laser fluencies used in the experiments. The imprint area on the silicon substrate corresponds to the laser irradiation spot. However, the continuity of the original film is not maintained, as in the case of the first configuration with an air gap. It should be also mentioned that the character of the metal transfer has noticeably changed; the position and size of the transferred large metal fragments are in good agreement with the damage to the metal on the donor surface (see Figure 4a,c). Furthermore, there are noticeably fewer small metal droplets compared to the previous experiments with a gap (Figure 2c). The fluence range for successful graphene transferring remains the same.

**Figure 5:**
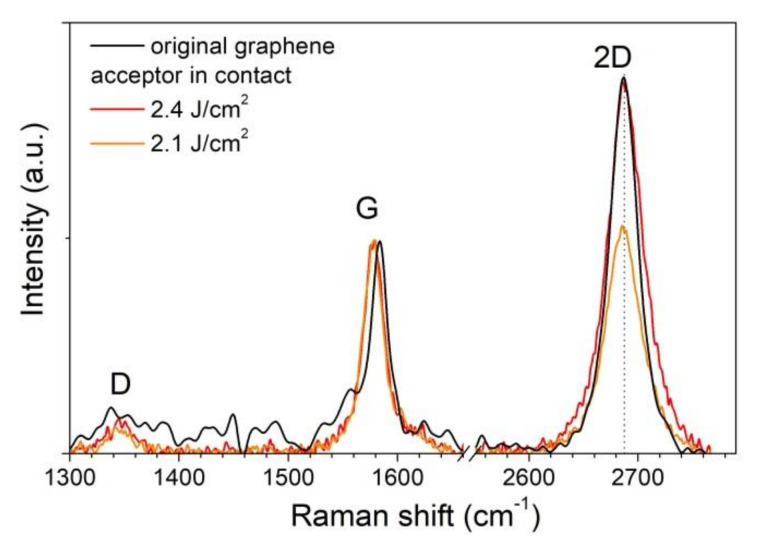
Raman spectra of the graphene films, recorded from the initial film on the donor substrate and areas on the acceptor substrates after the transfer procedure with zero distance between substrates.

An analysis of the material transferred in the scheme without a gap between the substrates by Raman spectroscopy (Figure 5) shows the preservation of the initial structure of the graphene film. No shift of the 2D peak to the lower wavenumber in the transferred film up to the threshold laser fluence is observed, in contrast to the film transferred in the scheme with a gap of 50 μm between the substrates. This indicates the absence of noticeable creasing and stress in the transferred film. In this case, optical images (see Figure 4c) demonstrate the same deposition of separate graphene flakes on a silicon substrate as in the absence of contact between the donor and acceptor (see Figure 2c). Additionally, a smaller change in the width and intensity ratio of the 2D and G peaks is observed in comparison with the spectra in Figure 3. After the transfer, the Id/Ig ratio increases from 0.07 for the initial film to 0.1 and 0.15 for the transferred graphene material with a fluence of 2.1 and 2.4 J/cm^2^, respectively. The increase in ratio corresponds to a decrease in the distance between defects from 38 to 32 and 26 nm, respectively.

**Figure 6:**
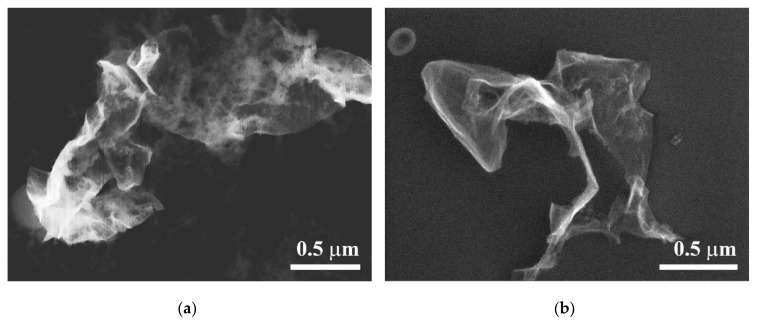
SEM images from individual flakes of single-layer graphene on the silicon substrate after the BB-LIFT procedure with a fluence of 2.1 J/cm^2^: (**a**) 50 μm gap between the donor and acceptor substrates; (**b**) zero gap between the substrates. Image (**b**) was taken with the acceptor surface inclined to the electron beam at 60 degrees.

For a more detailed comparison and analysis of the morphology of the transferred graphene in the two considered configurations of the laser transfer scheme, we used SEM images. Figure 6 shows images of separately transferred graphene flakes on silicon acceptor substrates after exposure with a fluence of 2.1 J/cm^2^, most promising for the imprint quality (Figure 4). For comparable areas of the transferred graphene flakes in Figure 6, the SEM image is less clear in the case of the transfer with a gap, which can indicate the surface charging during scanning by an electron beam. This can be caused by noticeably larger damage to the graphene film during laser transfer with a 50 μm gap between the substrates. The spongy surface of the graphene is noteworthy, i.e., the presence of multiple nano-ruptures of the surface, as well as a greater number of its local bends, is observed in Figure 6a, compared to graphene transferred in the scheme with the substrates in contact. The charge can also be drained by local oxidation of graphene during its heating and transfer, which was also mentioned in the analysis of the Raman spectra (Figure 3). In the case of the transfer without a gap, the transferred film oxidizes less before it comes into contact with the acceptor due to a zeroing or decrease in the time of flight and contact with ambient air, which improves the ohmic contact with the substrate. Another reason is the lower temperature of the transferred material in contact mode due to an increase in thermal conductivity. Thus, the decrease in structural damage of the transferred carbon film observed in Figure 6b explains the preservation of the initial spectral characteristics of the transferred graphene—there is no shift of the 2D peak in Figure 5. Nevertheless, even in the case of no gap, the transferred graphene flakes have a size of about 1 μm and are noticeably wrinkled. A possible cause of the creasing is the insufficient adhesion of graphene to the receiving substrate or its twisting on the donor substrate as a result of heating, before the contact with the acceptor. Figure 6b shows the surface image taken with the tilt of the silicon substrate relative to the electron beam. It demonstrates the absence of full contact between the transferred graphene flake and the surface of the silicon plate. The reason for the fragmentation of the initial film onto micron islands during the contact transfer can be the boiling of the adsorbate, which accumulates between the graphene and the hydrophilic metal of the donor substrate during exposure of the samples to air. The recent experiments on laser heating of graphene on SiO_2_ [28] have shown an important role of water adsorbate at the hydrophilic substrate/graphene interface in this process. Pulsed-laser-induced local heating to 200–300 °C can lead to the boiling of the adsorbed layers of water and the rupture of the polycrystalline graphene film along the boundaries. A decrease in the effect of graphene heating during laser transfer can be achieved by further increasing the thickness of the absorbing metal layer or by reducing the laser pulse duration. However, the demonstrated method in its present state can be used to print the crumpled graphene for the fabrication of arrays of electron emitters [29] or supercapacitors [30].

## 4. Conclusions

The BB-LIFT of the crumpled fragments of graphene film was demonstrated. The increase in the thickness of the absorbing metal layer allowed us to transfer the graphene material from all over the irradiated spot of the donor onto the acceptor substrate without signs of the metal. Reducing the gap between the substrates to a value of zero results in matching the patterns on the acceptor to the shape of the laser spot. Raman spectroscopy indicates the preservation of the initial structure of graphene after the LIFT, with a slight change in the distance between defects from 38 to 32 nm at the optimal conditions of the transfer. The SEM analysis shows the transfer of crumpling micrometer-scale islands of the graphene film. Therefore, the applied technique requires further optimization of the transfer conditions so as to print a continuous pixel from a graphene film.

## References

[1] Geim A.K., Novoselov K.S. (2007). The rise of graphene. Nat. Mater..

[2] Schwierz F. (2010). Graphene transistors. Nat. Nanotech..

[3] Novoselov K.S., Geim A.K., Morozov S.V., Jiang D., Zhang Y., Dubonos S.V., Grigorieva I.V., Firsov A.A. (2004). Electric field effect in atomically thin carbon films. Science.

[4] Wu Y., Jenkins K.A., Valdes-Garcia A., Farmer D.B., Zhu Y., Bol A.A., Dimitrakopoulos C., Zhu W., Xia F., Avouris P. (2012). State-of-the-Art Graphene High-Frequency Electronics. Nano Lett..

[5] Lin Y.-M., Valdes-Garcia A., Han S.-J., Farmer D.B., Meric I., Sun Y., Wu Y., Dimitrakopoulos C., Grill A., Avouris P. (2011). Wafer-Scale Graphene Integrated Circuit. Science.

[6] Berg Y., Zenou M., Dolev O., Kotler Z. (2015). Temporal pulses shaping for smoothing of printed metal surfaces. Opt. Eng..

[7] Grant-Jacob J.A., Mills B., Feinaeugle M., Sones C.L., Oosterhuis G., Hoppenbrouwers M.B., Eason R.W. (2013). Micron-scale copper wire printed using femtosecond laser-induced forward transfer with automated donor replenishment. Opt. Mater. Express.

[8] Serra P., Colina M., Fernandez-Pradas J.M., Sevilla L., Morenza J.L. (2004). Preparation of functional DNA microarrays through laser-induced forward transfer. Appl. Phys. Lett..

[9] Zergioti I., Karaiskou A., Papazoglou D.G., Fotakis C., Kapsetaki M., Kafetzopoulos D. (2005). Time resolved schlieren study of sub-picosecond and nanosecond laser transfer of biomaterials. Appl. Surf. Sci..

[10] Doraiswamy A., Narayan R.J., Lippert T., Urech L., Wokaun A., Nagel M., Hopp B., Dinescu M., Modi R., Auyeung R.C.Y. (2006). Excimer laser forward transfer of mammalian cells using a novel triazene absorbing layer. Appl. Surf. Sci..

[11] Thomas B., Alloncle A.P., Delaporte P., Sentis M., Sanaur S., Barret M., Collot P. (2007). Experimental investigations of laser induced forward transfer process of organic thin films. Appl. Surf. Sci..

[12] Palla-Papavlu A., Dinca V., Luculescu C., Shaw-Stewart J., Nagel M., Lippert T., Dinescu M. (2010). Laser induced forward transfer of soft materials. J. Opt..

[13] Kononenko T.V., Alloncle P., Konov V.I., Sentis M. (2009). Laser transfer of diamond nanopowder induced by metal film blistering. Appl. Phys. A.

[14] Smits E.C.P., Walter A., Leeuw D.M.D., Asadi K. (2017). Laser induced forward transfer of graphene. Appl. Phys. Lett..

[15] Kononenko T.V., Nagovitsyn I.A., Chudinova G.K., Mihailescu I.N. (2010). Application of clean laser transfer for porphyrin micropatterning. Appl. Surf. Sci..

[16] Kononenko T.V., Kamalov M.A., Popovich M.Y., Konov V.I., Sentis M.L. (2010). Choice of a target with metal coating for laser-induced transfer of ultradispersed materials. Quantum. Electron..

[17] Kononenko T.V., Alloncle P., Konov V.I., Sentis M. (2011). Shadowgraphic imaging of laser transfer driven by metal film blistering. Appl. Phys. A.

[18] Arutyunyan N.R., Komlenok M.S., Kononenko T.V., Dezhkina M.A., Popovich A.F., Konov V.I. (2019). Printing of single-wall carbon nanotubes via blister-based laser-induced forward transfer. Laser Phys..

[19] Dezhkina M.A., Komlenok M.S., Pivovarov P.A., Rybin M.G., Arutyunyan N.R., Popovich A.F., Obraztsova E.D., Konov V.I. (2020). Blister-based laser-induced forward transfer of 1D and 2D carbon nanomaterials. J. Phys. Conf. Ser..

[20] Chen X., Zhang L., Chen S. (2015). Large area CVD growth of the graphene. Synth. Metals.

[21] Rybin M.G., Islamova V.R., Obraztsova E.A., Obraztsova E.D. (2018). Modification of graphene electronic properties via controllable gas-phase doping with copper chloride. Appl. Phys. Lett..

[22] Barin G.B., Song Y., de Fátima Gimenez I., Souza Filho A.G., Barreto L.S., Kong J. (2015). Optimized graphene transfer: Influence of polymethylmethacrylate (PMMA) layer concentration and baking time on graphene final performance. Carbon.

[23] Blake P., Hill E.W., Castro Neto A.H. (2007). Making graphene visible. Appl. Phys. Lett..

[24] Ferrari A.C., Basko D.M. (2013). Raman spectroscopy as a versatile tool for studying the properties of graphene. Nat. Nanotechnol..

[25] Ferralis N. (2010). Probing mechanical properties of graphene with Raman spectroscopy. J. Mater. Sci..

[26] Gokus T., Nair R.R., Bonetti A., Bohmler M., Lombardo A., Novoselov K.S., Geim A.K., Ferrari A.C., Hartschuh A. (2009). Making graphene luminescent by oxygen plasma treatment. ACS Nano.

[27] Lucchese M.M., Stavale F., Martins Ferreira E.H., Vilani C., Moutinho M.V.O., Capaz R.B., Achete C.A., Jorio A. (2010). Quantifying ion-induced defects and Raman relaxation length in graphene. Carbon.

[28] Frolov V.D., Zavedeev E.V., Pivovarov P.A., Khomich A.A., Grigorenko A.N., Konov V.I. (2015). Water at the graphene–substrate interface: Interaction with short laser pulses. Quantum. Electron..

[29] Kleshch V.I., Bandurin D.A., Serbun P., Ismagilov R.R., Lutzenkirchen-Hecht D., Muller G., Obraztsov A.N. (2018). Field Electron Emission from CVD Nanocarbon Films Containing Scrolled Graphene Structures. Phys. Status Solidi B.

[30] Luo J., Jang H.D., Huang J. (2013). Effect of Sheet Morphology on the Scalability of Graphene-Based Ultracapacitors. ACS Nano.

